# Pre-treatment with the CDK4/6 inhibitor palbociclib improves the efficacy of paclitaxel in TNBC cells

**DOI:** 10.1038/s41598-019-49484-4

**Published:** 2019-09-10

**Authors:** Daniele Cretella, Claudia Fumarola, Mara Bonelli, Roberta Alfieri, Silvia La Monica, Graziana Digiacomo, Andrea Cavazzoni, Maricla Galetti, Daniele Generali, Pier Giorgio Petronini

**Affiliations:** 1 Department of Medicine and Surgery, University of Parma, Parma, Italy; 2 Italian Workers’ Compensation Authority (INAIL) Research Center, Parma, Italy; 3 Center of Excellence for Toxicological Research (CERT), Department of Medicine and Surgery, University of Parma, Parma, Italy; 4 Department of Medical, Surgery and Health Sciences, University of Trieste, Trieste, Italy; 5 Breast Cancer Unit, ASST, Cremona, Italy

**Keywords:** Breast cancer, Breast cancer

## Abstract

Triple Negative Breast Cancer (TNBC) is a challenging disease due to the lack of druggable targets; therefore, chemotherapy remains the standard of care and the identification of new targets is a high clinical priority. Alterations in the components of the cell cycle machinery have been frequently reported in cancer; given the success obtained with the CDK4/6 inhibitor palbocicib in ER-positive BC, we explored the potential of combining this drug with chemotherapy in Rb-positive TNBC cell models. The simultaneous combination of palbociclib with paclitaxel exerted an antagonistic effect; by contrast, the sequential treatment inhibited cell proliferation and increased cell death more efficaciously than single treatments. By down-regulating the E2F target *c*-myc, palbociclib reduced HIF-1α and GLUT-1 expression, and hence glucose uptake and consumption both under normoxic and hypoxic conditions. Importantly, these inhibitory effects on glucose metabolism were enhanced by palbociclib/paclitaxel sequential combination; the superior efficacy of such combination was ascribed to the ability of paclitaxel to inhibit palbociclib-mediated induction of AKT and to further down-regulate the Rb/E2F/*c*-myc signaling. Our results suggest that the efficacy of standard chemotherapy can be significantly improved by a pre-treatment with palbociclib, thus offering a better therapeutic option for Rb-proficient TNBC.

## Introduction

Dysregulation of the cell cycle is a hallmark of cancer that leads to aberrant cellular proliferation with the consequent increase of genome instability. The first cyclin dependent kinases (CDKs) activated in response to mitogenic stimulation are CDK4 and CDK6 that, after binding with cyclin D, promote retinoblastoma protein (Rb) hyperphosphorylation and inactivation with the consequent release of the transcription factor E2F, thus prompting cell cycle progression through the S phase.

The inhibition of cell cycle regulators such as CDK4 and CDK6 has become a new therapeutic frontier for the treatment of breast cancer (BC). To date, the CDK4/6 inhibitors palbociclib, ribociclib, and abemaciclib have been approved for the treatment of estrogen receptor (ER)-positive, human epidermal growth factor receptor 2 (HER2)-negative advanced or metastatic BC in combination with an endocrine therapy^1^. At present no validated biomarkers of sensitivity or resistance to CDK4/6 inhibitors have been identified, although the expression of a functional Rb protein has been suggested as a predictive factor of response^2^. Moreover, the expression of proteins involved in the control of cell cycle progression, such as cyclin D1, and the loss of the cell cycle inhibitor p16^INK4a^ have been associated with a better response to CDK4/6 inhibitors in BC cells^3,4^.

Triple negative breast cancer (TNBC) lacks the expression of ER, progesterone receptor (PR) and HER-2. This subtype accounts for approximately 15–20% of BC^5^ and is characterized by the poorest prognosis in respect to other BC malignancies. The chemotherapeutic regimen remains the only option for TNBC patients and the identification of new drug targets is urgently needed.

Although TNBC is a highly heterogenic disease^6^, alterations in the components of the cell cycle machinery have been frequently reported. For instance, *Rb* inactivation, due to mutation or homozygous loss of the gene, is observed in around 7–20% of TNBC^7,8^. In addition, the loss of p16^INK4^ occurs with high frequency in TNBC and has been correlated with the poor prognosis of this subtype^9^. An altered expression of cyclin D and E, CDK4/6, and CDK2 has also been observed in TNBC^10–12^. On the basis of these genetic features, TNBCs with a Rb-positive, p16^INK4^-negative profile might represent the subpopulation of TNBC suitable for the treatment with CDK4/6 inhibitors^4,13^. However, the simultaneous association between palbociclib and different chemotherapeutic agents has shown mainly an antagonistic effect^14,15^ due to the reduced sensitivity of non-cycling cells to chemotherapeutic drugs. Nonetheless, the timing and the sequence of drug exposure might play a critical role in drug activity, and the evaluation of different schedules of treatment may represent a new approach for the combination of palbociclib with chemotherapy.

Metabolic reprogramming of cancer cells is another hallmark of cancer with a role as a therapeutic target^16^. A relevant feature of palbociclib is its capability to inhibit glucose metabolism, as we previously showed in TNBC cell models^13^. The Rb/E2F/c-myc axis plays a role in the regulation of several metabolic processes, such as glucose production and glycolytic metabolism, indicating a close relationship between metabolic responses and proliferative stimuli^17^. In particular, the E2F pathway drives the cellular metabolism towards glycolysis, by inducing the expression of enzymes involved in the glycolytic process, such as phosphofructokinase, and by inhibiting the mitochondrial oxidative metabolism^18^.

In the present study we evaluated the potential of combining the CDK4/6 inhibitor palbociclib with chemotherapeutic agents currently used for the treatment of TNBC patients, such as paclitaxel, following different schedules of treatment (simultaneous versus sequential treatment). We demonstrated that the sequential treatment inhibited cell proliferation and induced cell death more efficaciously than single agent treatments. In addition, the impairment of glucose metabolism contributed to the efficacy of such combination.

## Results

### Effects of palbociclib in combination with chemotherapy

As we previously reported, both MDA-MB-231 and HCC38 TNBC cell lines displayed the molecular features associated with palbociclib sensitivity (Rb and cyclin D1 expression, loss of p16^INK4^); in accordance, palbociclib treatment inhibited cell proliferation in these cell models^13^.

**Figure 1:**
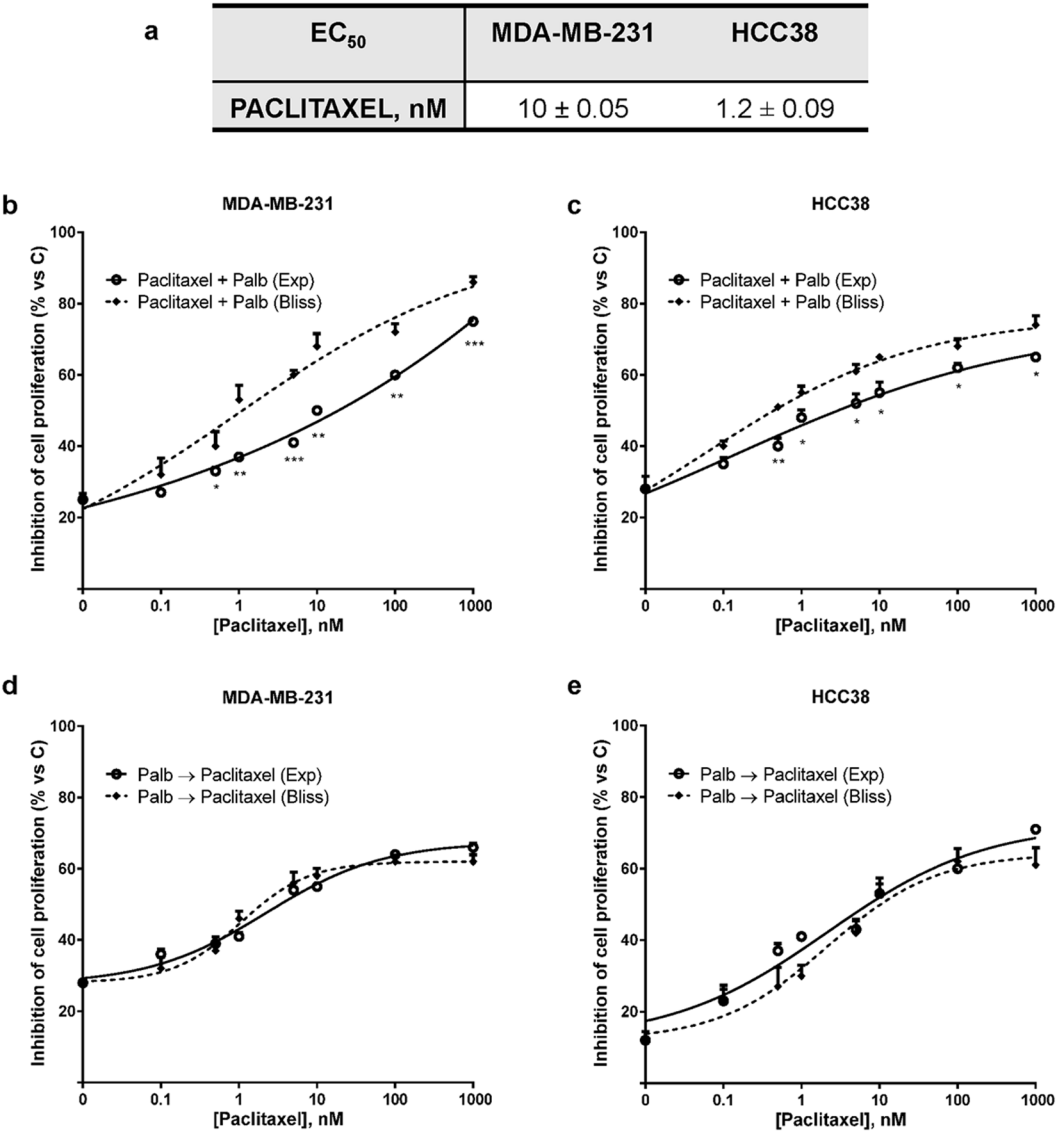
Palbociclib combined with paclitaxel induces different effects on cell proliferation depending on the treatment schedule. (**a**) MDA-MB-231 and HCC38 cells were treated with increasing concentrations of paclitaxel. After 72 h cell proliferation was evaluated by CV staining. Data are expressed as EC_50_ values and are means ± SD of three independent experiments. MDA-MB-231 and HCC38 cells were treated with 0.5 µM palbociclib alone or in combination with increasing concentrations of paclitaxel (**b**,**c**) or with 0.5 µM palbociclib for 24 h followed by exposure to increasing concentrations of paclitaxel alone for 48 h (**d**,**e**). After 72 h cell proliferation was assessed by CV assay. The effect of the drug combinations was evaluated using the Bliss interaction model. Data are mean values ± SD of three independent experiments.

**Figure 2:**
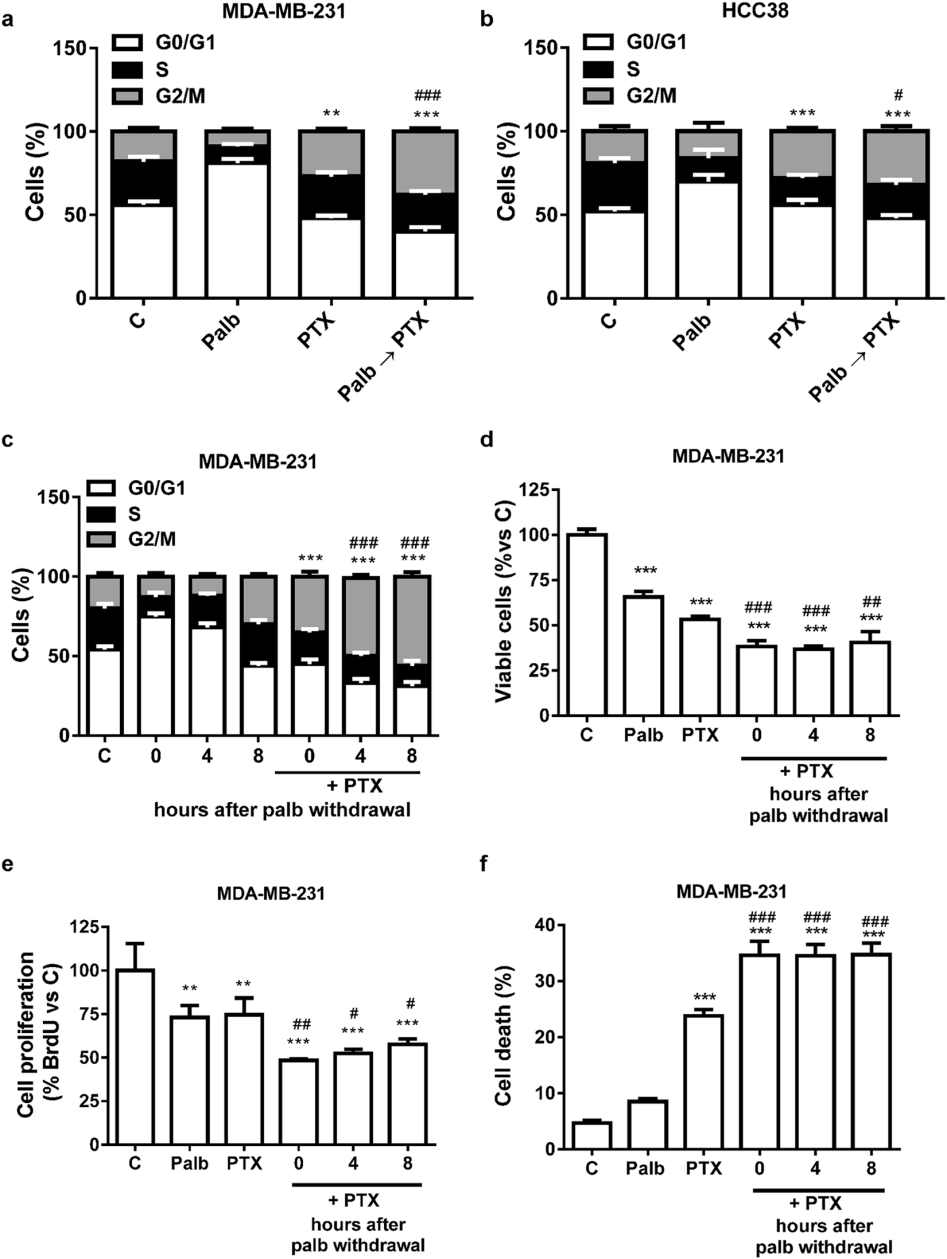
Palbociclib-pre-treatment induces a G1-cell cycle phase synchronization and the sequential treatment with paclitaxel enhances the percentage of cells in G2/M phase. MDA-MB-231 (**a**) and HCC38 (**b**) cells were incubated for 48 h with 0.5 µM palbociclib (Palb) and 10 nM paclitaxel (PTX) alone or simultaneously combined (Palb + PTX), or were treated with palbociclib for 24 h and then with paclitaxel for further 24 h (Palb → PTX). Then the cells were stained with PI and the distribution of cells in cell cycle phases was determined by flow cytometry. Results are representative of three independent experiments. **p < 0.01, ***p < 0.001 vs G2/M C; ^#^p < 0.05, ^###^p < 0.001 vs G2/M PTX. **c** MDA-MB-231 cells were treated with 0.5 µM palbociclib for 24 h followed by an incubation in palbociclib-free medium for 0, 4 or 8 h (recovery). At the end of the recovery, the cells were treated with 10 nM paclitaxel for 24 h. After PTX treatment, the cells were stained with PI and the distribution of cells in cell cycle phases was determined by flow cytometry. Results are representative of three independent experiments. ***p < 0.001 vs G2/M 0-4-8 h; ^###^p < 0.001 vs G2/M 0 h + PTX. MDA-MB-231 cells were treated as in **c** and then cell viability (**d**), cell proliferation (**e**), and cell death (**f**) were assessed after 48 h of paclitaxel treatment by counting the cells in a Bürker hemocytometer with the trypan blue exclusion method, by BrDU incorporation and by fluorescence microscopy after Hoechst 33342/PI staining, respectively. Results are representative of at least two independent experiments.***p < 0.001 vs C; ^##^p < 0.01, ^###^p < 0.001 vs PTX.

Therefore, MDA-MB-231 and HCC38 cell lines were used to investigate the effects of palbociclib in combination with paclitaxel, a chemotherapeutic drug currently used for the treatment of TNBC. After showing that both TNBC cell lines were sensitive to paclitaxel (Fig. 1a), two different schedules of combination were tested: a simultaneous and a sequential treatment. In the first schedule, MDA-MB-231 and HCC38 cells were treated with increasing concentrations of paclitaxel in association with a fixed concentration of palbociclib (Fig. 1b,c). Through the Bliss experimental model, we demonstrated that the simultaneous combinations gave rise to an antagonistic effect. This antagonism can be ascribed to the reduced activity of cytotoxic chemotherapeutic agents, directed against cycling cells, when used in cells already arrested in G0/G1 phase, as previously suggested^15^. The second approach was based on a sequential treatment; in particular, MDA-MB-231 and HCC38 cells were treated with palbociclib for 24 h, then palbociclib was removed and the cells were exposed to paclitaxel for further 48 h. As demonstrated by the Bliss experimental model (Fig. 1d,e), this schedule of treatment produced additive inhibitory effects on cell proliferation. Similar results on the inhibition of cell proliferation were obtained under hypoxic conditions (data not shown). Also the association with 5-fluorouracil produced comparable results (Supplementary Fig. S1). In contrast with the simultaneous treatment, the efficacy of such regimen is likely due to synchronized cell re-entry into cell cycle upon palbociclib removal^19^, which renders the cells more susceptible to chemotherapeutic agents active in S or G2/M phase. Indeed, after palbociclib treatment around 80% and 70% of cells (for MDA-MB-231 and HCC38, respectively) were blocked in the G1 phase, whereas paclitaxel increased the proportion of cells in G2/M phase (around 30% in both cell lines); the sequential treatment further increased this percentage, reaching ~40% in both cell models (Fig. 2a,b).

To evaluate whether a period of recovery following palbociclib removal could further improve the effectiveness of paclitaxel, MDA-MB-231 cells were pre-incubated with palbociclib for 24 h, then paclitaxel was added immediately, 4 h or 8 h after palbociclib withdrawal. As shown in Fig. 2c, after palbociclib removal the cells progressively re-entered the cell cycle and the percentage of cells arrested in the G0/G1 phase decreased, whereas the number of cells within the S and G2/M cell cycle phases increased. After 8 h of drug withdrawal, the normal distribution of cells in each cell cycle phase was completely restored. When paclitaxel was added after 4 or 8 h of palbociclib withdrawal, a higher percentage of cells accumulated in G2/M phase in comparison with no recovery. However, the sequential combination reduced the number of viable cells more efficaciously than the single treatments independently of the time elapsed between the two treatments, suggesting that the timing of paclitaxel addition after palbociclib removal is not critical for its efficacy (Fig. 2d). To demonstrate that the reduced cell viability was effectively associated with the inhibition of cell proliferation, we performed a BrdU incorporation assay. As shown in Fig. 2e, not only palbociclib, as expected, but also paclitaxel reduced the proportion of cells engaged in DNA synthesis as compared to control, and the drug sequential combination further decreased this percentage. In addition, paclitaxel, and even more the combination, induced a substantial increase in cell death (Fig. 2f).

**Figure 3:**
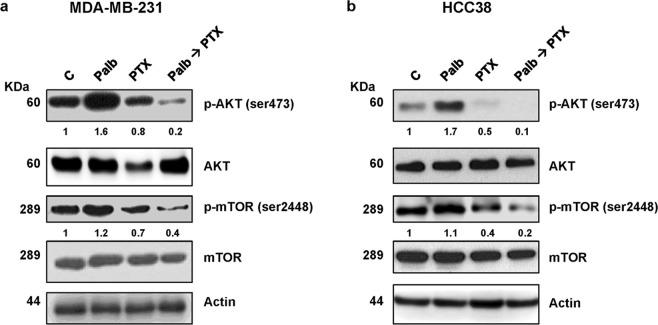
The sequential treatment inhibits palbociclib-mediated induction of the PI3K/mTOR pathway. MDA-MB-231 (**a**) and HCC38 (**b**) cell lines were incubated for 48 h with 0.5 µM palbociclib and 10 nM paclitaxel, alone or in sequential treatment (Palb → PTX). The expression of the indicated proteins was analyzed by Western blotting. The immunoreactive spots were quantified by densitometric analysis and p-AKT/AKT and p-mTOR/mTOR ratios were calculated; the data are expressed as fold increase versus control (control value = 1). Results are representative of two independent experiments.

As previously demonstrated^13^, palbociclib treatment up-regulated the phosphorylation levels of AKT and the downstream target mTOR in both MDA-MB-231 and HCC38 cells (Fig. 3a,b). In contrast, paclitaxel alone was able to reduce the basal levels of p-AKT and p-mTOR expression and most importantly inhibited palbociclib-mediated induction of this pathway, an effect likely contributing to the efficacy of the sequential treatment.

**Figure 4:**
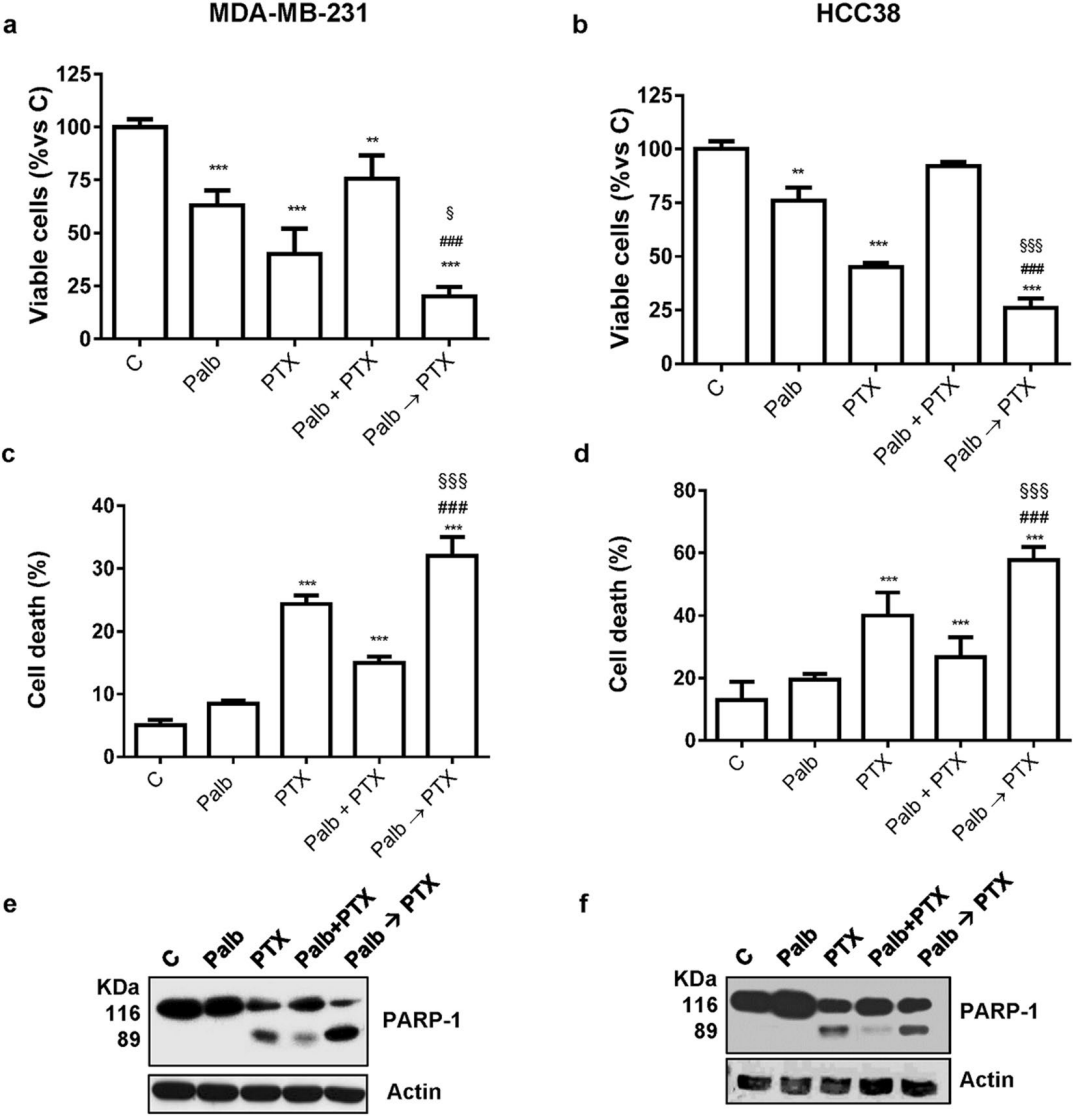
Palbociclib combined with paclitaxel induces different effects on cell death depending on the treatment schedule. TNBC cell lines were incubated for 48 h with 0.5 µM palbociclib and 10 nM paclitaxel, alone or simultaneously combined (Palb + PTX), or were treated with palbociclib for 24 h and then with paclitaxel for further 48 h (Palb → PTX). Cell viability (**a**,**b**) and cell death (**c**,**d**) were assessed by counting the cells in a Bürker hemocytometer with the trypan blue exclusion method and by fluorescence microscopy after Hoechst 33342/PI staining, respectively. **p < 0.01, ***p < 0.001 vs C; ^###^p < 0.001 vs PTX; ^§^p < 0.05, ^§§§^p < 0.001 vs Palb + PTX. (**e**,**f**) PARP-1 cleavage was detected by Western blotting. Data in (**a**–**d**) are mean values ± SD of three independent experiments. Results in (**e** and **f**) are representative of two independent experiments.

We also evaluated the effects of the different treatment schedules on cell death. No relevant signs of death were detected in MDA-MB-231 and HCC38 cells treated with palbocilcib alone, confirming its cytostatic effects. However, as previously shown, palbociclib treatment followed by exposure to paclitaxel reduced cell viability (Fig. 4a,b), inducing a significant increase of cell death in comparison with either agents alone (Fig. 4c,d). In contrast, after the simultaneous treatment with palbociclb and paclitaxel, cell viability was less affected, thus confirming the antagonistic effect of this schedule. Indeed, in this condition, the incorporation of BrdU was not reduced (Supplementary Fig. S2) and a lower induction of cell death was observed.

Paclitaxel-mediated cell death was associated with cleavage of the caspase substrate PARP-1 (Fig. 4e,f). Interestingly, the expression of the cleaved 89 KDa peptide was further increased when paclitaxel treatment was preceded by the incubation with palbociclib; in contrast, the simultaneous drug treatment resulted in a slight induction of PARP-1 cleavage.

### The sequential treatment of palbociclib followed by paclitaxel impaired glucose energy metabolism

**Figure 5:**
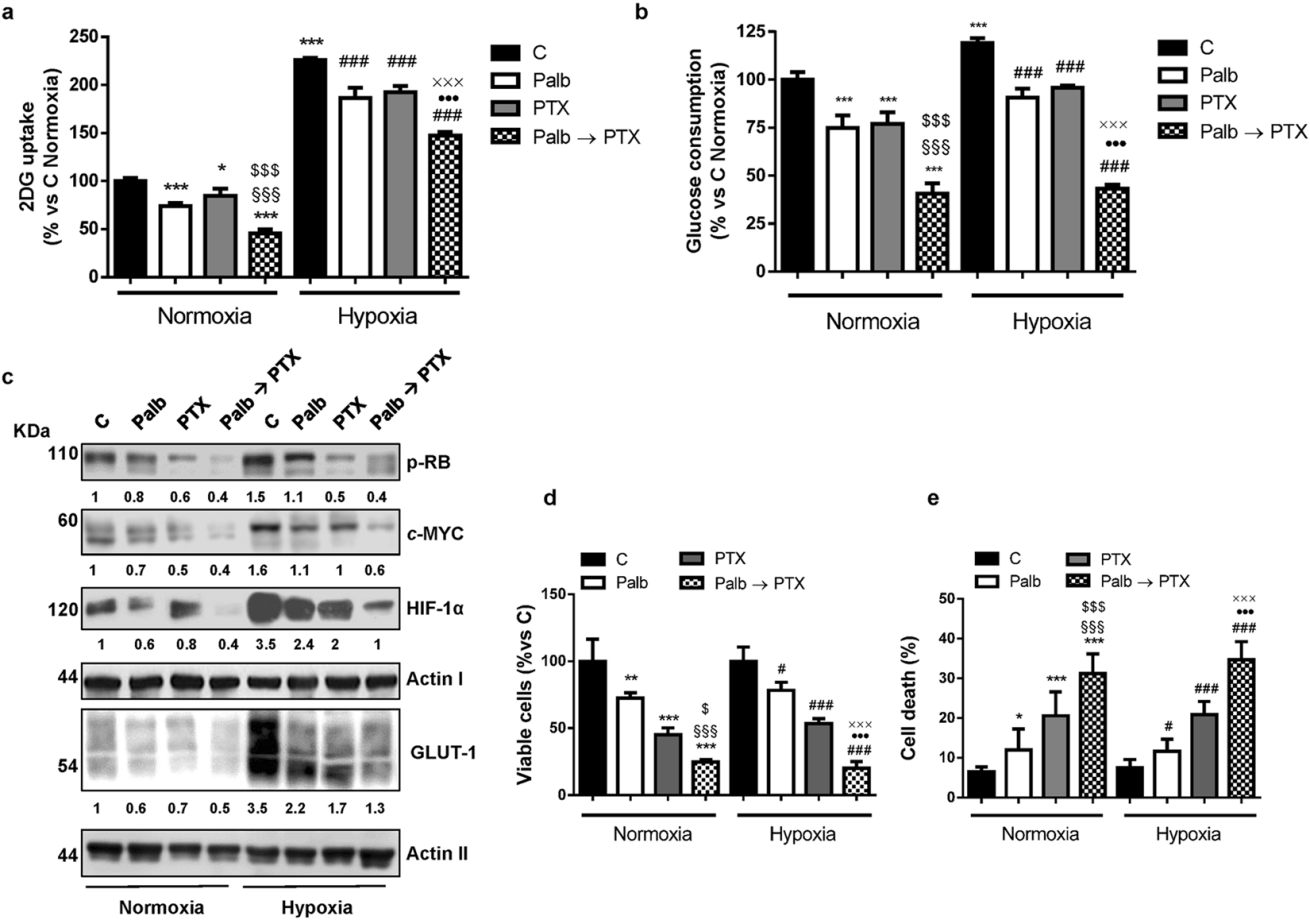
Sequential treatment of palbociclib followed by paclitaxel hinders glucose metabolism under both normoxic and hypoxic conditions. MDA-MB-231 cells were treated for 48 h with 0.5 µM palbociclib and 10 nM paclitaxel alone or treated with palbociclib for 24 h and then with paclitaxel for further 24 h (Palb → PTX) under normoxic or hypoxic (1% O_2_) conditions. Glucose uptake (**a**) and glucose consumption (**b**) were measured. *p < 0.05, ***p < 0.001 vs C Normoxia (N); ^§§§^p < 0.001 vs Palb N; ^$$$^p < 0.001 vs PTX N; ^###^p < 0.001 vs C Hypoxia (H); ^●●●^p < 0.001 vs Palb H; ^xxx^p < 0.001 vs PTX H. **c** The expression of the indicated proteins was analyzed by Western blotting. The immunoreactive spots were quantified by densitometric analysis and the ratios between each protein and the corresponding Actin, were calculated; the data are expressed as fold increase versus control (control value = 1). Cell viability (**d**) and cell death (**e**) were assessed by counting the cells in a Bürker hemocytometer with the trypan blue exclusion method and by fluorescence microscopy after Hoechst 33342/PI staining, respectively. Data in (**a**,**b**,**d**,**e**) are mean values ± SD of three independent experiments. Results in **c** are representative of two independent experiments.

**Figure 6:**
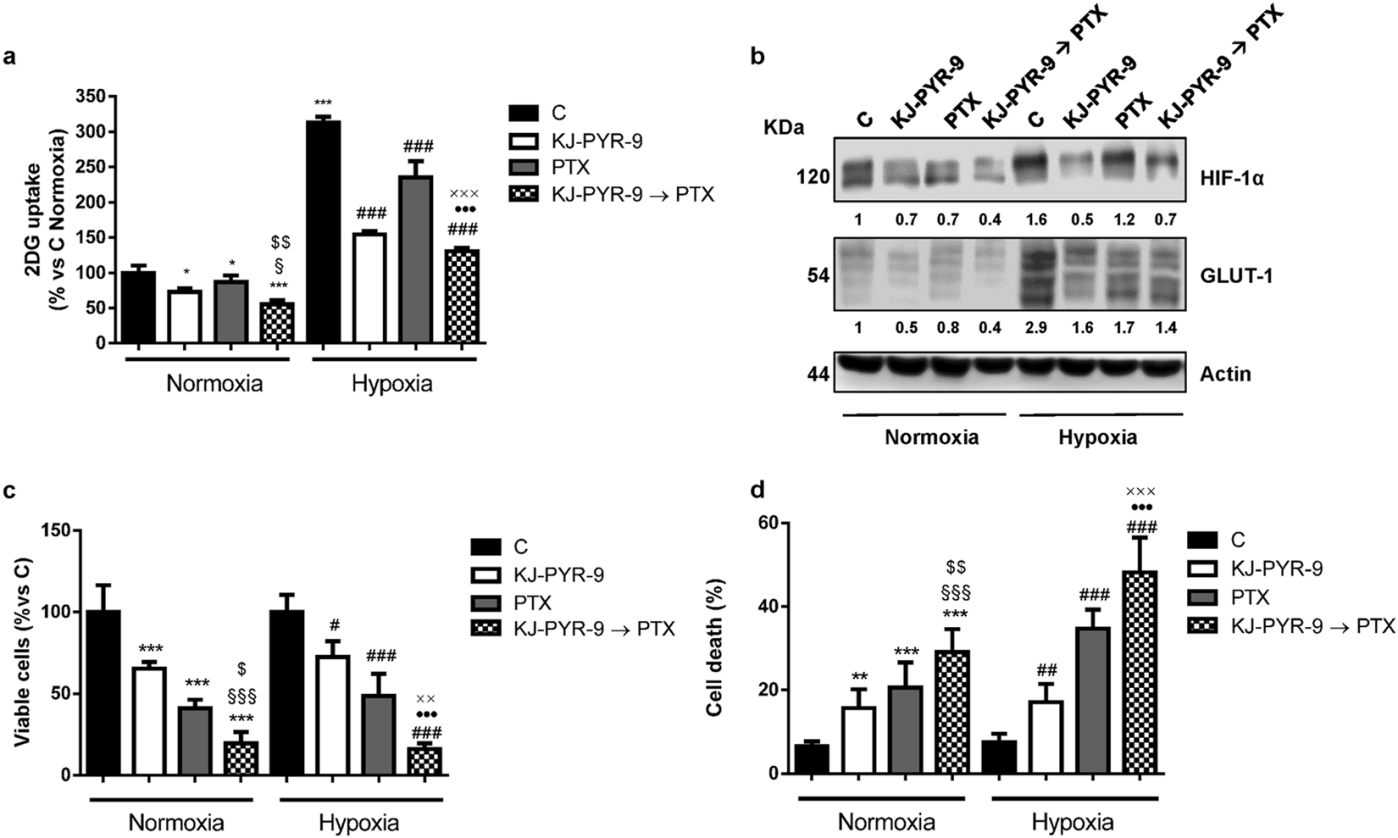
Inhibition of *c*-myc activity affects glucose metabolism in TNBC cells under both normoxic and hypoxic conditions. MDA-MB-231 cells were treated for 48 h with 20 µM KJ-PYR-9 and 10 nM paclitaxel alone or treated with KJ-PYR-9 for 24 h and then with paclitaxel for further 24 h (KJ-PYR-9 → PTX) in normoxic or hypoxic (1% O_2_) conditions. Glucose uptake (**a**) was measured. **b** The expression of the indicated proteins was analyzed by Western blotting. The immunoreactive spots were quantified by densitometric analysis, and HIF-1α/Actin and GLUT-1/Actin ratios were calculated; the data are expressed as fold increase versus control (control value = 1). Cell viability (**c**) and cell death (**d**) were assessed by counting the cells in a Bürker hemocytometer with the trypan blue exclusion method and by fluorescence microscopy after Hoechst 33342/PI staining, respectively. *p < 0.05, ***p < 0.001 vs C Normoxia (N); ^§^p < 0.05, ^§§§^p < 0.001 vs KJ-PYR-9 N; ^$^p < 0.05, ^$$^p < 0.01, ^$$$^p < 0.001 vs PTX N; ^#^p < 0.05, ^##^p < 0.01, ^###^p < 0.001 vs C Hypoxia (H); ^●●●^p < 0.001 vs KJ-PYR-9 H; ^xx^p < 0.01, ^xxx^p < 0.001 vs PTX H. Data in (**a**,**c**,**d**) are mean values ± SD of three independent experiments. Results in (**b**) are representative of two independent experiments.

Cell cycle-related proteins have been demonstrated to play a role in the regulation of cell energy metabolism^18^, and we previously showed that palbociclib hampered glucose metabolism in MDA-MB-231 cells^13^. This prompted us to evaluate the impact of the sequential treatment of palbociclib followed by paclitaxel on glucose metabolism, under both normoxic and hypoxic conditions. As shown in Fig. 5a,b, both palbociclib and paclitaxel reduced the glucose uptake and consumption in MDA-MB-231 cells, by down-regulating the expression of HIF-1α and GLUT-1 glucose transporter (Fig. 5c); these effects were further enhanced by the sequential combination. Exposure to hypoxic conditions induced the expression of HIF-1α and GLUT-1 (Fig. 5c), thus promoting a shift towards glycolysis, which was hindered by the sequential combination more efficaciously than single treatments. The inhibition of cell proliferation as well as the induction of apoptosis observed after drug treatment in hypoxic condition were comparable to those obtained under normoxia (Fig. 5d,e). Interestingly, the expression of p-Rb and the E2F-dowstream target *c*-myc was reduced not only by palbociclib, as previously demonstrated^13^, but also by paclitaxel, and even more by the sequential treatment (Fig. 5c). Considering the critical role that *c*-myc plays in the regulation of cell metabolism^20^, the stronger inhibition of *c*-myc expression may provide a mechanistic explanation to the superior efficacy of the combination in inhibiting glucose metabolism over the individual treatments. To better investigate the involvement of *c*-myc down-regulation in the impairment of glucose metabolism, we performed a sequential treatment using the myc inhibitor KJ-PYR-9 instead of palbociclib in MDA-MB-231 cells. As shown in Fig. 6a, this inhibitor reduced the glucose uptake under both normoxic and hypoxic conditions, as a consequence of HIF-1α and GLUT-1 down-regulation (Fig. 6b); most importantly, the sequential combination with paclitaxel potentiated these effects. Therefore, KJ-PYR-9 was able to recapitulate palbociclib effects, reinforcing the suggestion that the negative impact of palbociclib on glucose metabolism is actually mediated by *c*-myc inhibition. Further corroborating these results the observation that proliferation of MDA-MB-231 cells was slowed down by KJ-PYR-9 and that an even stronger inhibition was achieved when treatment with this drug was followed by exposure to paclitaxel (Fig. 6c). Similarly, cell death was enhanced after the sequential drug treatment (Fig. 6d).

## Discussion

In this study, we provide evidence that the sequential treatment of palbociclib followed by paclitaxel is effective in TNBC cell lines, producing additive anti-proliferative and pro-apoptotic effects associated with impairment of glucose metabolism.

We previously showed that palbociclib inhibited cell proliferation in TNBC cell lines characterized by Rb, cyclin D1, and CDK6 protein expression, along with p16^INK4^ loss^13^. In addition, we demonstrated that a superior efficacy was achieved when palbociclib was combined with PI3K/mTOR inhibitors following a sequential combined approach. The present study confirms our previous suggestion that palbociclib treatment may be effective not only in ER-positive breast cancer, but also in Rb-proficient TNBC. Most importantly, our findings suggest that palbociclib may be considered for TNBC treatment as a valuable tool to improve the efficacy of chemotherapy, which remains the standard of care for this type of BC, given its extreme aggressiveness and the lack of well-established targets.

In this regard, a clear notion emerging from our results is that a combinatorial strategy with CDK4/6 inhibitors and chemotherapy must be designed carefully, due to the potential interference between the cytostatic action of CDK4/6 inhibitors and the cytotoxic mechanisms underlying the activity of chemotherapeutics acting on cycling cells, thus corroborating previous findings^15,21^. Indeed, when palbociclib was given simultaneously with paclitaxel or 5-FU, an antagonistic effect emerged, which may discourage the clinical use of such combination. However, when palbociclib was exploited as a pre-treatment for chemotherapy, we demonstrated an additive inhibitory effect on cell proliferation associated with a significant increase in apoptotic cell death. The efficacy of this schedule relies on the reversible action of palbociclib on cell cycle: upon palbociclib removal, the cells arrested in G1 phase synchronously reenter the cell cycle in S phase, becoming more sensitive to the cytotoxic effect of chemotherapy. Such an approach was previously used to sensitize myeloma cells to bortezomib-induced apoptosis^22^. Similarly, sarcoma cells were shown to be more sensitive to agents active in S-G2 phase, such as doxorubicin and Wee1 kinase inhibitors, after palbociclib pre-treatment^21^. It is worth to note that, in contrast with our findings, palbociclib was efficaciously combined with paclitaxel following a concurrent regimen in multidrug resistant ovarian cancer cells^23^. In addition, a staggered regimen in which treatment with taxanes preceded exposure to palbociclib demonstrated enhanced anti-tumor effects in squamous cell lung cancer cell models^24^. Altogether, these results suggest that the choice of the drug delivery schedule may depend on the type of cancer and is therefore a critical aspect requiring careful consideration when planning CDK4/6 inhibitors plus chemotherapy-based therapies. This notion finds confirmation in the results from a phase I trial demonstrating the safety and preliminary efficacy of palbociclib/paclitaxel combination in Rb-positive BC, regardless of subtype^19^. In this study, an alternating schedule was followed, with palbociclib treatment preceding paclitaxel administration in order to allow G1-synchronization and subsequent cell cycle re-entry upon palbociclib removal. It is worth noting that paclitaxel was administered after a few drug-free days. In MDA-MB-231 cells, the duration of the recovery period after palbociclib removal did not affect the responsiveness to paclitaxel, although it is conceivable that *in vivo* a prolonged recovery is required to maximize the increase in the proportion of cells in S/M phase, therefore potentiating subsequent paclitaxel activity.

The anti-proliferative and pro-apoptotic effects of the sequential combination of palbociclib and paclitaxel were associated with the ability of paclitaxel to enhance the downregulation of the Rb/E2F pathway and to inhibit palbociclib-mediated induction of the AKT/mTOR pathway. Importantly, these mechanisms also affected glucose metabolism contributing to the cytotoxicity of the combination.

Altered energy metabolism is considered as one of the hallmarks of cancer, supporting the acquisition and maintenance of malignant properties^25^. Multiple oncogenic signaling pathways play a role in cancer metabolic reprogramming, suggesting that their selective targeting may produce anti-tumor effects also by tackling on energy metabolism^26^.

A variety of evidence indicate that the CDK4/Rb/E2F pathway promotes a coordinate modulation of cell cycle progression and energy metabolism by directly contributing to the control of metabolic processes, such as lipid synthesis and glycolysis^18^. In a previous study we demonstrated that palbociclib impairs glucose metabolism in TNBC cells by inhibiting GLUT-1 and HIF-1α expression, glucose uptake and accumulation under both normoxic and hypoxic conditions^13^. Here we confirm that the underlying mechanism involves the inhibition of *c*-myc protein, whose expression is under E2F control^27^. Indeed, the small molecule KJ-PYR-9, which inhibits *c*-myc function by interfering with MYC-MAX complex formation^28^, was able to recapitulate palbociclib-mediated inhibitory effects on glucose metabolism. Interestingly, KJ-PYR-9 also down-regulated HIF-1α under normoxic and hypoxic conditions, suggesting that the reduced accumulation of HIF-1α promoted by palbociclib depended on the inhibition of *c*-myc expression. Deregulated *c*-myc and HIF-1α cooperate to increase glucose metabolism through the induction of glycolytic enzymes, thus providing adaptive advantages to cancer cells in the hypoxic tumor microenvironment^29^. Importantly, overexpressed myc has been shown to regulate HIF-1α post-transcriptionally, inducing the expression of HIF-1α gene targets not only under hypoxia but also under normoxic conditions^30^. Therefore, *c*-myc down-regulation associated with palbociclib treatment in myc-overexpressing MDA-MB-231 cells may lead to impairment of glucose metabolism either directly or by inhibiting HIF-1α stabilization and accumulation.

In our study, also paclitaxel down-regulated p-Rb, *c*-myc, and HIF-1α expression under normoxia or hypoxia. This effect was enhanced by pre-treatment with either palbociclib or the myc inhibitor KJ-PYR-9, resulting in a greater reduction of GLUT-1 expression and in a further decrease of glucose uptake and consumption. In this context, it is worth noting that paclitaxel was also able to inhibit palbociclib-dependent induction of AKT. The inhibitory effect of paclitaxel on AKT phosphorylation was previously shown in ovarian cancer cells^31^. Moreover, the PI3K/AKT signaling has been involved in the cell cycle control through the regulation of cyclin D1, p-Rb, and E2F expression^32,33^. Therefore, inhibition of AKT by paclitaxel may account for the down-regulation of p-Rb and consequently *c*-myc protein.

The impact of palbociclib/paclitaxel combinations on glucose metabolism may be relevant in the clinic, considering that ^18^F-FDG PET/CT, largely used for both cancer staging and assessment of response to chemotherapy, has recently emerged as an useful tool for monitoring the response to palbociclib in ER-positive and HER2-negative metastatic BC patients, especially to select the patients with no clinical benefit^34^.

## Methods

### Cell culture

MDA-MB-231 and HCC-38 triple negative breast cancer cell lines were cultured in RPMI supplemented with 2 mM glutamine, 10% fetal bovine serum (FBS) and 100 U/ml penicillin, 100 μg/ml streptomycin.

Cells were purchased from the American Type Culture Collection (Manassas, VA), which authenticates the phenotypes of these cell lines on a regular basis (http://www.lgcstandards-atcc.org). Hypoxic conditions were established by placing the cells in a tissue culture incubator with controlled O_2_ levels (Binder GmbH, Tuttlingen, Germany).

### Drug treatment

Palbociclib (PD-0332991) was provided by Pfizer (New York City, NY); paclitaxel and 5-Fluorouracil (5-FU) were obtained from the inpatient pharmacy of University Hospital of Parma; KJ-PYR-9 was purchased from Calbiochem - Merck KGaA (Darmstadt, Germany). Palbociclib was dissolved in water. Chemotherapeutic drugs were dissolved in DMSO, and DMSO concentration never exceeded 0.1% (v/v); equal amounts of the solvent were added to control cells.

### Analysis of cell proliferation, cell viability/death and cell cycle

Quantification of cell proliferation was determined using a colorimetric immunoassay, based on the measurement of BrdU incorporation during DNA synthesis (#QIA58 – BrdU Cell Proliferation Assay, Calbiochem EMD Millipore Corporation, MA). The assay was performed according to the manufacturer’s instructions. Briefly, 10^4^ cells/well were incubated in 96 multiwell plates with 20 µl/well of 1:2000 BrdU solution during the last 16 h of the drug treatments. Then, the cells were denatured with a Fixative/Denaturing Solution and incubated for 60 min with 1:100 diluted mouse anti-BrdU mAbs conjugated to peroxidase. After removing the antibody, a substrate solution was added for 15 min and the reaction was stopped by adding a stop solution. The absorbance was measured at 450 nm with a reference wavelength at 570 nm using a microplate reader. Data were calculated as incorporated BrdU/mg protein and expressed as percent vs control.

Cell viability was evaluated by counting the cells in a Bürker hemocytometer with trypan blue exclusion method and by Crystal Violet (CV) staining as previously described^35^. The nature of the interaction between palbociclib and chemotherapy was calculated using the Bliss additivism model as previously described^36^. A theoretical dose-response curve was calculated for combined inhibition using the equation EBliss = EA + EB − EA * EB, where EA and EB are the percent of inhibition versus control obtained by paclitaxel or 5-FU (A) and palbociclib (B) alone and the EBliss is the percent of inhibition that would be expected if the combination was exactly additive. If the combination effect is higher than the expected EBliss value the interaction is synergistic, while if the effect is lower, the interaction is antagonistic. Otherwise, the effect is additive and there is no interaction between drugs.

Cell death was analyzed as described elsewhere^37^. Distribution of the cells in the cell cycle was determined by propidium iodide (PI) staining and flow cytometry analysis as previously described^38^.

### Western blotting

The procedures for protein extraction, solubilization, and protein analysis by western blotting are described elsewhere^39^. Antibodies against p-Rb^Ser780^ (#9307), c-Myc (#9402), PARP-1 (#9532), p-AKT^Ser473^ (#9271), AKT (#9272), p-mTOR^Ser2448^ (#2971), mTOR (#2972), were from CST (Danvers, MA); anti-GLUT-1 (ab40084) was from Abcam (Cambridge, UK). Antibody against HIF-1α (#610959) was from BD Biosciences (Franklin Lakes, NJ). Anti-β-actin was from BioVision (#3598) (Milpitas, CA). Horseradish peroxidase-conjugated secondary antibodies and chemiluminescence system were from Millipore (Millipore, MA). Reagents for electrophoresis and blotting analysis were from BIO-RAD Laboratories (Hercules, CA). The chemiluminescent signal was acquired by C-DiGit® Blot Scanner and the spots were quantified by Image Studio™ Software, LI-COR Biotechnology (Lincoln, NE).

### Glucose uptake and consumption

Glucose uptake was measured as described previously^13^, calculated as pmol of 2-deoxy-D-glucose (2DG)/mg protein/5 min and expressed as percent vs control condition. Glucose consumption was determined using a Glucose (HK) Assay Kit (Sigma-Aldrich)^13^ and calculated subtracting the glucose amount in the spent media to glucose in cell-free media. Data were calculated as mg glucose/mg protein and expressed as percent vs control.

### Statistical analysis

Statistical analyses were carried out using GraphPad Prism 6.00 software. Statistical significance of differences among data was estimated by two-tailed Student’s t test. Comparison among groups was made using analysis of variance (one-way ANOVA, repeated measures) followed by Tukey’s post-test.

## Supplementary information

Supplementary Fig. 1

Supplementary Fig. 2

Supplementary Informations

## Data Availability

The datasets generated during and/or analysed during the current study are available from the corresponding author on reasonable request.
