# Supplementary material for: Pre-treatment with the CDK4/6 inhibitor palbociclib improves the efficacy of paclitaxel in TNBC cells
Source: Sci Rep. 2019 Sep 10;9:13014. doi: 10.1038/s41598-019-49484-4 (PMC6736958; doi:10.1038/s41598-019-49484-4)
Supplement: Supplementary file 1 — Supplementary Fig. 1 [file 41598_2019_49484_MOESM1_ESM.pdf]

Pre-treatment with the CDK4/6 inhibitor palbociclib improves the efficacy of paclitaxel in TNBC cells

Daniele Cretella+, Claudia Fumarola+, Mara Bonelli*, Roberta Alfieri, Silvia La Monica, Graziana Digiacomo, Andrea Cavazzoni, Maricla Galetti, Daniele Generali, Pier Giorgio Petronini.

*Correspondence to:
Mara Bonelli - Department of Medicine and Surgery, University of Parma, Italy
Tel +39 0521 903766
E mail mara.bonelli@unipr.it

a

EC_{50}	MDA-MB-231	HCC38
**5-FU, μM**	7 ± 0.03	2 ± 0.03

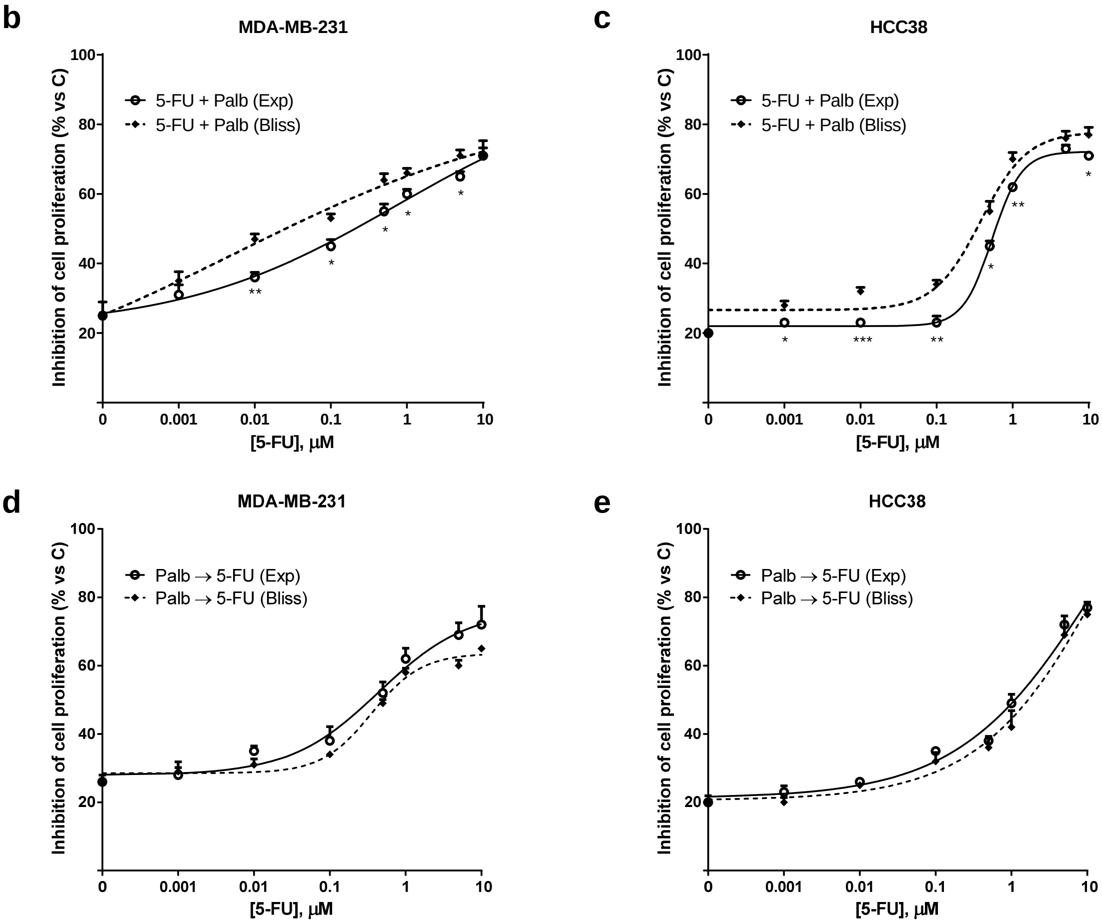

Supplementary Fig.1 Palbociclib combined with 5-FU induces different effects on cell proliferation depending on the treatment schedule

a MDA-MB-231 and HCC38 cells were treated with increasing concentrations of 5-FU. After 72h cell proliferation was evaluated by CV staining. Data are expressed as EC50 values and are means ±SD of three independent experiments. MDA-MB-231 and HCC38 cells were treated with 0.5 µM palbociclib alone or in combination with increasing concentrations of 5-FU (**b**, **c**) or with 0.5 µM palbociclib for 24h followed by exposure to increasing concentrations of 5-FU alone for 48h (**d**, **e**). After 72h cell proliferation was assessed by CV assay. The effect of the drug combinations was evaluated using the Bliss interaction model. Data are mean values ±SD of three independent experiments.
