# Supplementary material for: Pre-treatment with the CDK4/6 inhibitor palbociclib improves the efficacy of paclitaxel in TNBC cells
Source: Sci Rep. 2019 Sep 10;9:13014. doi: 10.1038/s41598-019-49484-4 (PMC6736958; doi:10.1038/s41598-019-49484-4)
Supplement: Supplementary file 2 — Supplementary Fig. 2 [file 41598_2019_49484_MOESM2_ESM.pdf]

Pre-treatment with the CDK4/6 inhibitor palbociclib improves the efficacy of paclitaxel in TNBC cells

Daniele Cretella+, Claudia Fumarola+, Mara Bonelli*, Roberta Alfieri, Silvia La Monica, Graziana Digiacomo, Andrea Cavazzoni, Maricla Galetti, Daniele Generali, Pier Giorgio Petronini.

*Correspondence to:
Mara Bonelli - Department of Medicine and Surgery, University of Parma, Italy
Tel +39 0521 903766
E mail mara.bonelli@unipr.it

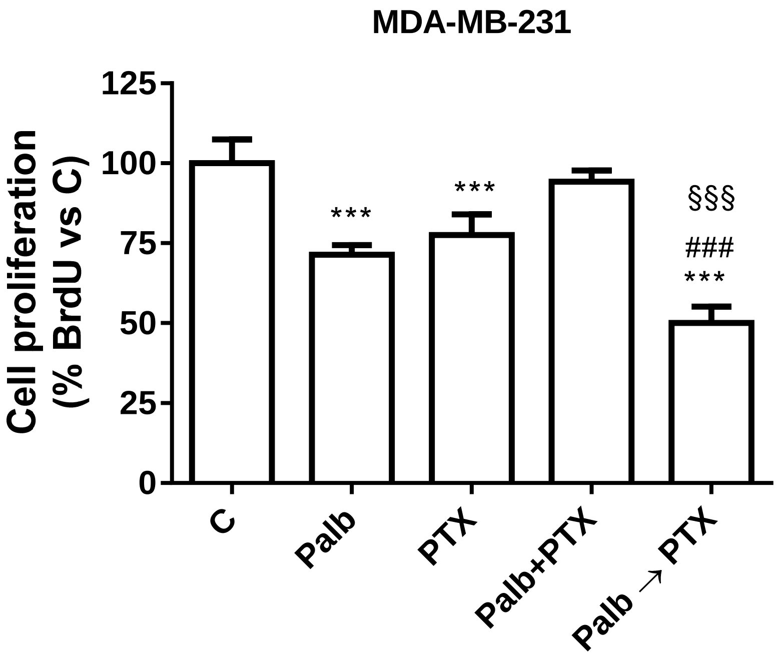

Supplementary Fig.2 Palbociclib combined with paclitaxel induces different effects on cell proliferation depending on the treatment schedule

MDA-MB-231 cells were incubated for 48h with 0.5 μM palbociclib (Palb) and 10 nM paclitaxel (PTX) alone or simultaneously combined (Palb+PTX), or were treated with palbociclib for 24h and then with paclitaxel for further 48h (Palb→PTX). During the last 16h of treatment, BrdU was added and cell proliferation was assessed. Data are expressed as % of Brdu vs C and are representative of two independent experiments. ***$p<0.001$ vs C; ###$p<0.001$ vs PTX; §§§$p<0.001$ vs Palb+PTX.
