# Supplementary material for: Pre-treatment with the CDK4/6 inhibitor palbociclib improves the efficacy of paclitaxel in TNBC cells
Source: Sci Rep. 2019 Sep 10;9:13014. doi: 10.1038/s41598-019-49484-4 (PMC6736958; doi:10.1038/s41598-019-49484-4)
Supplement: Supplementary file 3 — Supplementary Informations [file 41598_2019_49484_MOESM3_ESM.pdf]

Pre-treatment with the CDK4/6 inhibitor palbociclib improves the efficacy of paclitaxel in TNBC cells

Daniele Cretella[+], Claudia Fumarola[+], Mara Bonelli[*], Roberta Alfieri, Silvia La Monica, Graziana Digiacomo, Andrea Cavazzoni, Maricla Galetti, Daniele Generali, Pier Giorgio Petronini.

*Correspondence to:
Mara Bonelli - Department of Medicine and Surgery, University of Parma, Italy
Tel +39 0521 903766
E mail mara.bonelli@unipr.it

Full Original Blots 1

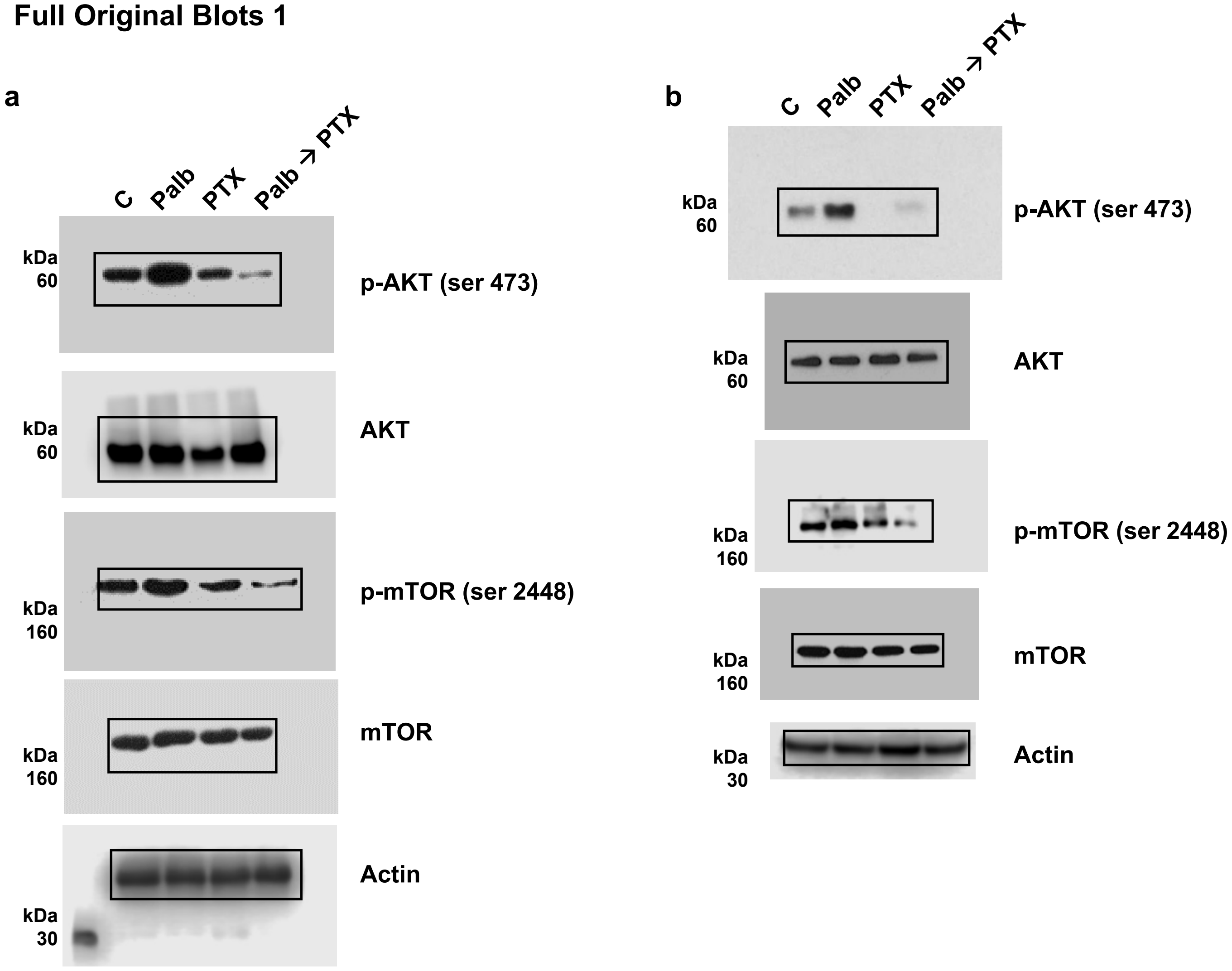

Full original blots of MDA-MB-231 (**a**) and HCC38 (**b**) cells used for Fig.3a,b. Based on the standard band positions, each blot membrane was cut into three pieces, which were then incubated with the corresponding antibodies. As reported in the Methods Section, the chemiluminescent signal was acquired by C-DiGit® Blot Scanner. After 12 minutes of acquisition, the images with the best balance between brightness/contrast were chosen, saved in jpeg format with 300 dpi of resolution and cropped as reported. The identification of the bands was based on the expected molecular weight.

Full Original Blots 2

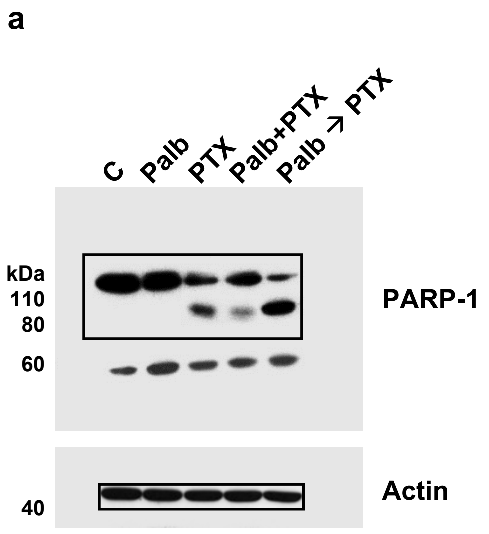

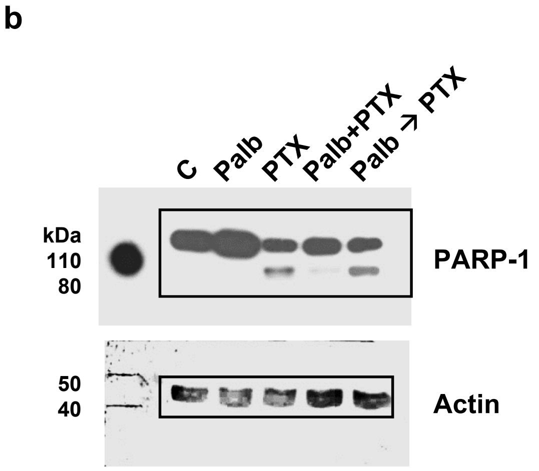

Full original blots of MDA-MB-231 (**a**) and HCC38 (**b**) cells used for Fig.4e,f. Based on the standard band positions, each blot membrane was cut into two pieces, which were then incubated with antibodies against PARP-1 or Actin. As reported in the Methods Section, the chemiluminescent signal was acquired by C-DiGit® Blot Scanner. After 12 minutes of acquisition, the images with the best balance between brightness/contrast were chosen, saved in jpeg format with 300 dpi of resolution and cropped as reported. The identification of the bands was based on the expected molecular weight.

Full Original Blots 3

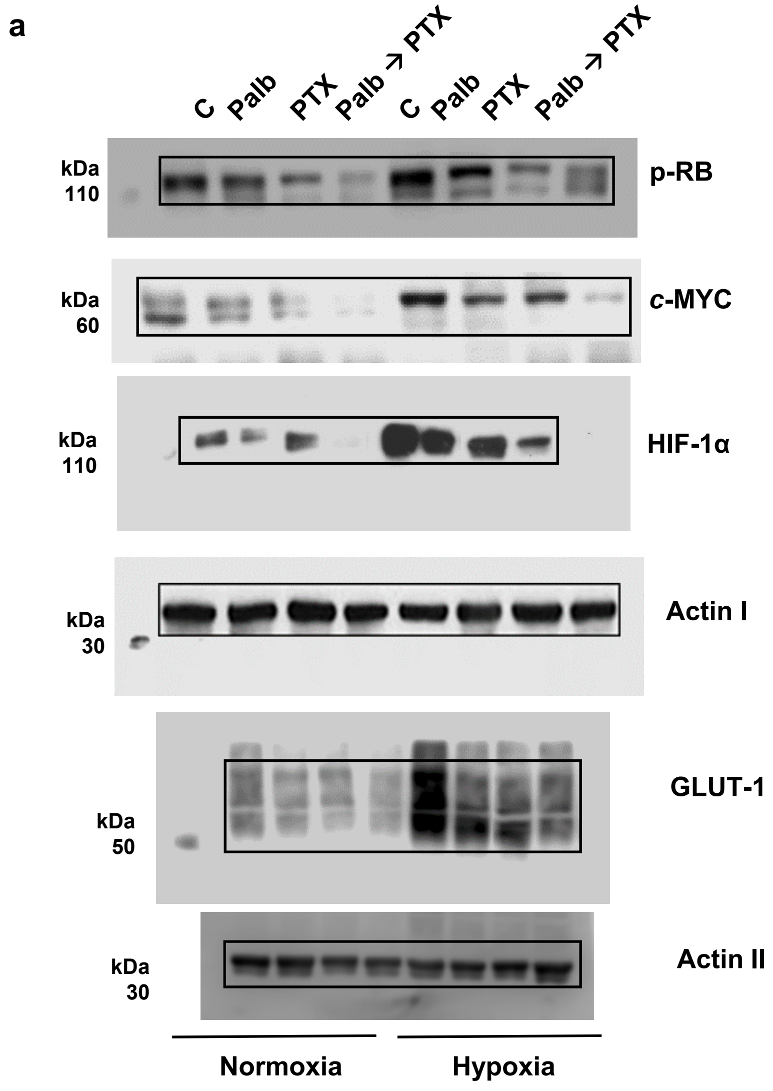

Full original blots of MDA-MB-231 cells used for Fig.5c. Based on the standard band positions, each blot membrane was cut into three pieces, which were then incubated with the corresponding antibodies. As reported in the Methods Section, the chemiluminescent signal was acquired by C-DiGit® Blot Scanner. After 12 minutes of acquisition, the images with the best balance between brightness/contrast were chosen, saved in jpeg format with 300 dpi of resolution and cropped as reported. The identification of the bands was based on the expected molecular weight.

Full Original Blots 4

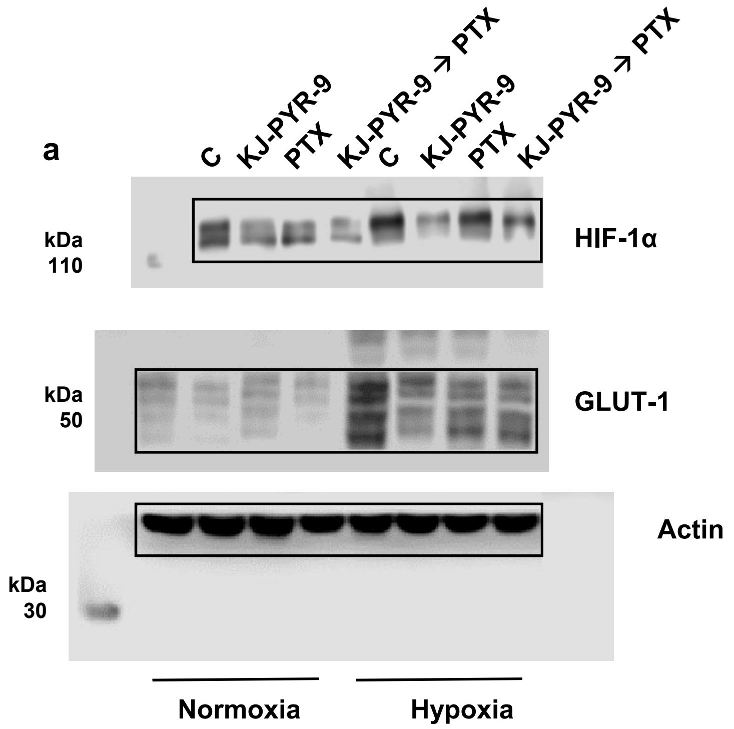

Full original blots of MDA-MB-231 cells used for Fig.6b. Based on the standard band positions, the blot membrane was cut into three pieces, which were then incubated with the corresponding antibodies. As reported in the Methods Section, the chemiluminescent signal was acquired by C-DiGit® Blot Scanner. After 12 minutes of acquisition, the images with the best balance between brightness/contrast were chosen, saved in jpeg format with 300 dpi of resolution and cropped as reported. The identification of the bands was based on the expected molecular weight.
